# Predicting instances of pathway ontology classes for pathway integration

**DOI:** 10.1186/s13326-019-0202-8

**Published:** 2019-06-13

**Authors:** Lucy Lu Wang, G. Thomas Hayman, Jennifer R. Smith, Monika Tutaj, Mary E. Shimoyama, John H. Gennari

**Affiliations:** 1 Department of Biomedical Informatics and Medical Education, University of Washington, 850 Republican St, Seattle, 98109 WA USA; 2 Department of Biomedical Engineering, Medical College of Wisconsin, 8701 W Watertown Plank Rd, Milwaukee, 53226 WI USA

**Keywords:** Pathway ontology, Ontology-based data integration, Semi-automated ontology curation, Ontology mapping, Pathway data interoperability

## Abstract

**Background:**

To improve the outcomes of biological pathway analysis, a better way of integrating pathway data is needed. Ontologies can be used to organize data from disparate sources, and we leverage the Pathway Ontology as a unifying ontology for organizing pathway data. We aim to associate pathway instances from different databases to the appropriate class in the Pathway Ontology.

**Results:**

Using a supervised machine learning approach, we trained neural networks to predict mappings between Reactome pathways and Pathway Ontology (PW) classes. For 2222 Reactome classes, the neural network (NN) model generated 10,952 class recommendations. We compared against a baseline bag-of-words (BOW) model for predicting correct PW classes. A 5% subset of Reactome pathways (111 pathways) was randomly selected, and the corresponding class recommendations from both models were evaluated by two curators. The precision of the BOW model was higher (0.49 for BOW and 0.39 for NN), but the recall was lower (0.42 for BOW and 0.78 for NN). Around 78% of Reactome pathways received pertinent recommendations from the NN model.

**Conclusions:**

The neural predictive model produced meaningful class recommendations that assisted PW curators in selecting appropriate class mappings for Reactome pathways. Our methods can be used to reduce the manual effort associated with ontology curation, and more broadly, for augmenting the curators’ ability to organize and integrate data from pathway databases using the Pathway Ontology.

## Background

Ontologies can be used to align and integrate data from multiple sources. In the case of biological pathways, there are numerous databases collecting and describing information about pathway networks, but no centralized schema to organize these various pathways. A shared organizational scheme would allow researchers to identify semantically similar pathways, providing a framework for pathway data integration.

Pathways are a form of graph data describing biological function. Individual pathway modules describe the interactions between dozens or hundreds of genes, proteins, and molecules, and how these interactions contribute to events of biological consequence. The complexities of analyzing genomic data have led to a rise in the use of pathways for pathway analysis, a class of statistical methods that aggregate single gene effects over the genes described in pathway modules. These pathway analysis techniques (such as gene set enrichment analysis (GSEA) [1] or network-based pathway analysis methods [2]) allow variations in gene expression to be interpreted at a functional level. Due to the large variety of pathways available from different databases, pathway analysis often leverages pathways from multiple databases. For example, MSigDB, which is often used as a source of gene sets for GSEA, combines pathways from the Kyoto Encyclopedia of Genes and Genomes (KEGG), the National Cancer Institute’s Pathway Interactions Database (NCI-PID), and Reactome [3].

Combining pathways from different databases results in redundancy in the pathway data set. The same or a similar pathway may be represented in multiple databases. Meta-resources such as Pathway Commons [4] and ConsensusPathDB [5] allow for querying and access to pathways from different databases, but lack the ability to collapse redundant pathways between databases. Other resources such as PathCards [6] or ReCiPa [7] use statistical methods to detect gene overlap between two pathways, merging pathways with significant overlapping entities into superpathways to reduce membership redundancy. However, these methods fail to retain the functional boundaries of pathways, which are crucial for pathway analysis result interpretation, i.e., allowing gene expression differences to be aggregated and interpreted at a functional level.

Pathways from different databases are challenging to integrate due to content and representational differences between various pathway databases. Previous studies have described the differences that exist between pairs of pathway databases [8–11], and in our prior work, we have categorically summarized ways in which pathway representations have been found to differ between many common pathway databases [12]. Although most databases provide data in pathway file sharing standards such as BioPAX [13], SBML [14], or GPML [15], these standards are insufficient for ensuring interoperability. Even when two databases present data using the same standard language, the different decisions of pathway editors at both individual and database levels can result in variable pathway representation [12].

Ontologies have been used successfully to combine disparate datasets in the biomedical domain [16–18]. We hypothesize that an ontology of pathway classes can be used to organize data from different pathway databases, allowing us to merge data while maintaining an understanding of the semantic relationships between various pathways. The Pathway Ontology (PW), an ontology of pathway terms, can be used as an anchoring ontology to identify similar pathways [19]. The PW was developed as part of the Rat Genome Database (RGD) as a means to catalog and describe the relationships among various biological pathways. The ontology covers broad pathway categories such as metabolic, regulatory, signaling, disease, and drug pathways, and allows for the representation of both subclass and mereological hierarchies via the *subclass* and *part-of* relationships respectively. The PW is a suitable ontology for integrating pathway data because it provides:

- a hierarchy of pathway classes and their relations to one another,
- classes describing altered and disease pathways, and
- existing mappings to pathways from KEGG, NCI-PID, and the Small Molecule Pathway Database (SMPDB).

The Gene Ontology (GO) describes biological processes, and could be a suitable ontology for pathway data integration based on its more developed classes and richer annotations [20]. However, the GO lacks classes describing altered or disease pathways, which are essential for downstream applications of pathway resources. The PW describes both altered and disease pathways in its class hierarchy and is therefore suitable for integrating pathway data.

Using the PW, we can group together semantically and functionally similar pathways by mapping them to the appropriate PW class. All pathways mapped to a particular PW class can then be merged together to form a normalized pathway representation of that class. This set of normalized pathways can be used in pathway analysis applications, and will have less redundancy compared to naively combined pathway datasets, as well as increased functional interpretability due to the preserved PW class hierarchy.

To better enable pathway data integration, we must map the content of other pathway databases to the PW. However, manual mappings are both laborious and time-consuming to produce. In light of limited curatorial resources, we propose a method to integrate computational prediction into the curation pipeline, allowing a predictive model to reduce the number of manual comparisons that need to be made by PW curators. Machine learning methods have been used with success for ontology-related tasks such as ontology learning, ontology completion, and ontology alignment [21, 22]. Rule-based techniques have been very successful, but supervised or semi-supervised approaches can also be used when training data are available. We propose and implement a supervised learning framework for inferring mappings between pathways from pathway databases and the PW, with a goal of reducing the hours associated with manual curation.

In this article, we describe efforts to generate PW class mappings for pathways from Reactome, one of the largest and most comprehensive pathway databases [23]. Our methods are generalizable to other pathway databases, such as BioCyc [24] and WikiPathways [25], that are not currently represented in the PW. We have applied our trained model to BioCyc and WikiPathways to generate mappings. Our contributions are two-fold; we introduce:

- A curation pipeline that integrates a predictive model with manual curation, and an evaluation of our prediction results, and
- Newly predicted and curated mappings between the PW and Reactome

In this work, we describe the design and implementation of this curation pipeline, with emphasis on our supervised mapping prediction model. We describe how mappings are generated and provide an evaluation of the results compared to a baseline bag-of-words (BOW) model. PW curators manually review a randomly selected subset of mapping outputs to determine the precision and recall of each model. We also discuss new mappings and relationships that we plan to add to the PW in future versions, with particular emphasis on expanding the *part-of* hierarchy and the inclusion of regulatory relationships through the usage of terms from the Relation Ontology.

By integrating a machine learning predictive model into the PW curation pipeline, we hope to reduce the burden of manual curation on our efforts to integrate pathway data. It is our hope that other researchers can incorporate similar methodology into their ontology curation pipelines, thereby reducing curatorial labor while increasing high quality mappings between datasets and ontologies.

## Methods

Our goal is to associate pathway instances from various databases to the correct class in the Pathway Ontology. The following describes our methods as applied to the Reactome database. Specifically, we map each Reactome pathway to a matching class in the PW if a matching class exists. In cases where no matching class exists, a new PW class is introduced to account for the pathway; the new class is inserted where appropriate into the PW class hierarchy.

Each class in the PW consists of its unique identifier and its descriptive information: a canonical name, aliases (synonyms), definition, and its location in the PW *subclass* and *part-of* hierarchies. Each Reactome pathway has similar descriptive information, along with the pathway content itself: the entities and relationships that describe the biochemical functions of the pathway. These pieces of descriptive information can be used to associate pathways with PW classes. Our goal is to build a predictive model leveraging this information along with training data to generate high-quality mapping recommendations between Reactome and the PW. This predictive model can then be inserted into the PW curation pipeline to improve the speed and quality of curated mappings. For this task, we propose a supervised machine learning algorithm that learns features and weights from the information provided for each PW class or Reactome pathway.

**Fig. 1:**
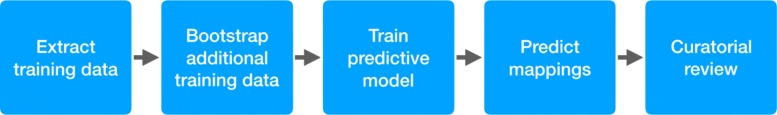
Semi-automated curation pipeline

The pipeline (Fig. 1) we propose and test consists of the following steps:

- Extract training data from the PW and the Unified Medical Language System (UMLS) Metathesaurus [26]
- Bootstrap additional training data by predicting high likelihood mappings between Reactome pathways and PW classes
- Train a neural network model using all training data
- Predict Reactome mappings to the PW using trained model
- Review predicted mappings manually for correctness and inclusion into the PW

We treat the predictive task as a binary classification problem, where given a pathway and a PW class, we predict whether the two have a high likelihood of matching. We construct two models, one which predicts matches over the names and aliases of pathways and PW classes, and one which predicts matches over the natural language definitions of pathways and PW classes. The distinction is introduced because not all pathways or PW classes have natural language definitions, and neural network models can be challenged by the presence of null fields in cases where training datasets are small. A subsequent decision module then collects the predictive model outputs for the separate name and definition models and combines these to form a final predicted similarity score.

Details for each step in the curation pipeline are provided in the following sections. We also provide a description of the candidate selector module we used for both negative data sampling and candidate selection when running the predictive model. All results presented discuss pathways from Reactome v65, released 2018, June 12.

### Baseline bag-of-words model

A bag-of-words (BOW) model is provided as a baseline model for comparison. This baseline is based on string similarity, and is similar to the way curators previously retrieved potential class matches for pathway instance annotation. For the BOW model, each pathway and PW class is represented as a set of word and *n*-gram tokens, generated from its names, definition, and the names of its parent and children classes. A *idf*-weighted Jaccard index is computed between the token set of a Reactome pathway (*A*) and the token set of a PW class (*B*) as:

1 $$ J_{weighted} = \frac{\sum_{t \epsilon A \cap B} idf(t)}{\sum_{t \epsilon A \cup B} idf(t)}  $$

For each Reactome pathway, PW classes with weighted Jaccard indices above a threshold similarity score are selected as output. The optimal threshold was determined using a grid search over the training data. All results provide comparisons between our neural network-based predictive model against this baseline model.

### Candidate selection

The candidate selector module takes in a pathway and outputs a ranked list of PW classes that are potential matches. Good matches are determined by large lexical overlap in descriptive information. We first generate a string representation of each pathway or PW class by appending together its names, definitions, and the names of all its parents and children. Each pathway string or PW class string from this corpus is then parsed to a set of word tokens and character *n*-gram tokens. Each token is weighted by its inverse document frequency (*idf*) in the entire corpus. Tokens with higher *idf* occur less frequently and may be more relevant for determining matches. The overall lexical overlap score between a pathway and a PW class is determined by summing the *idf* of all overlapping tokens between the two.

The candidate selector is used to reduce the number of necessary comparisons when predicting PW class mappings. When the candidate selector is given a pathway as input, it first selects all PW classes with *any* token overlap with the input pathway. The selector then sorts the overall lexical overlap scores for these PW classes and returns the top 20 as candidates. Instead of performing *m* comparisons for each pathway (where *m* is the number of PW classes), the candidate selector reduces the number of comparisons to 20.

The candidate selector is also used to generate “hard” negatives (see “Training data” section), which are negative training data where there is substantial lexical overlap between the pathway string and PW class string. “Hard” negatives are selected from the candidate list while ensuring no overlap with positive training data. Hard negatives are introduced into the training data to force greater predictive precision.

### Training data

To train a binary classifier, we require both positive and negative training data. Prior mappings of KEGG, NCI PID, and the SMPDB to the PW can be used as positive labeled training data. Together, 860 mappings are provided in the PW. These mappings exist over 732 unique PW classes, out of a total of 2627 classes; in other words, around 28% of PW classes have existing mappings to pathways. These mappings reference 206 unique pathways from KEGG, 76 from NCI-PID, and 557 from SMPDB.

For each PW class, negative mappings are also sampled from these three pathway databases for training. Approximately two “easy” and two “hard” negatives are sampled for each PW class, where “easy” negatives are randomly selected from the pathway database, and “hard” negatives are selected using the candidate selector module. Care was taken to ensure that no extracted negatives overlap with any positive training examples.

To augment these existing mappings, we also extract mappings from the UMLS Metathesaurus between Gene Ontology (GO) biological process terms and the Medical Subject Headings (MeSH) [26]. GO biological process classes overlap with concepts in the pathway space, and we believe these mappings can provide reasonable distant supervision for our classifier. From UMLS, we extract 732 mappings between MeSH and GO.

The breakdown of all extracted training data is given in Table 1. Of these, 860 positive and 7116 negative mappings are extracted from the PW and 732 positive and 325 negative mappings from the UMLS Metathesaurus.

**Table 1:** Training data by source

Source	No. positive	No. negative
PW mappings to KEGG, NCI-PID, and SMPDB	860	7116
GO/MeSH mappings	732	325
Bootstrapped PW/Reactome mappings	730	720
Total	2322	8161

### Bootstrapping

**Fig. 2:**
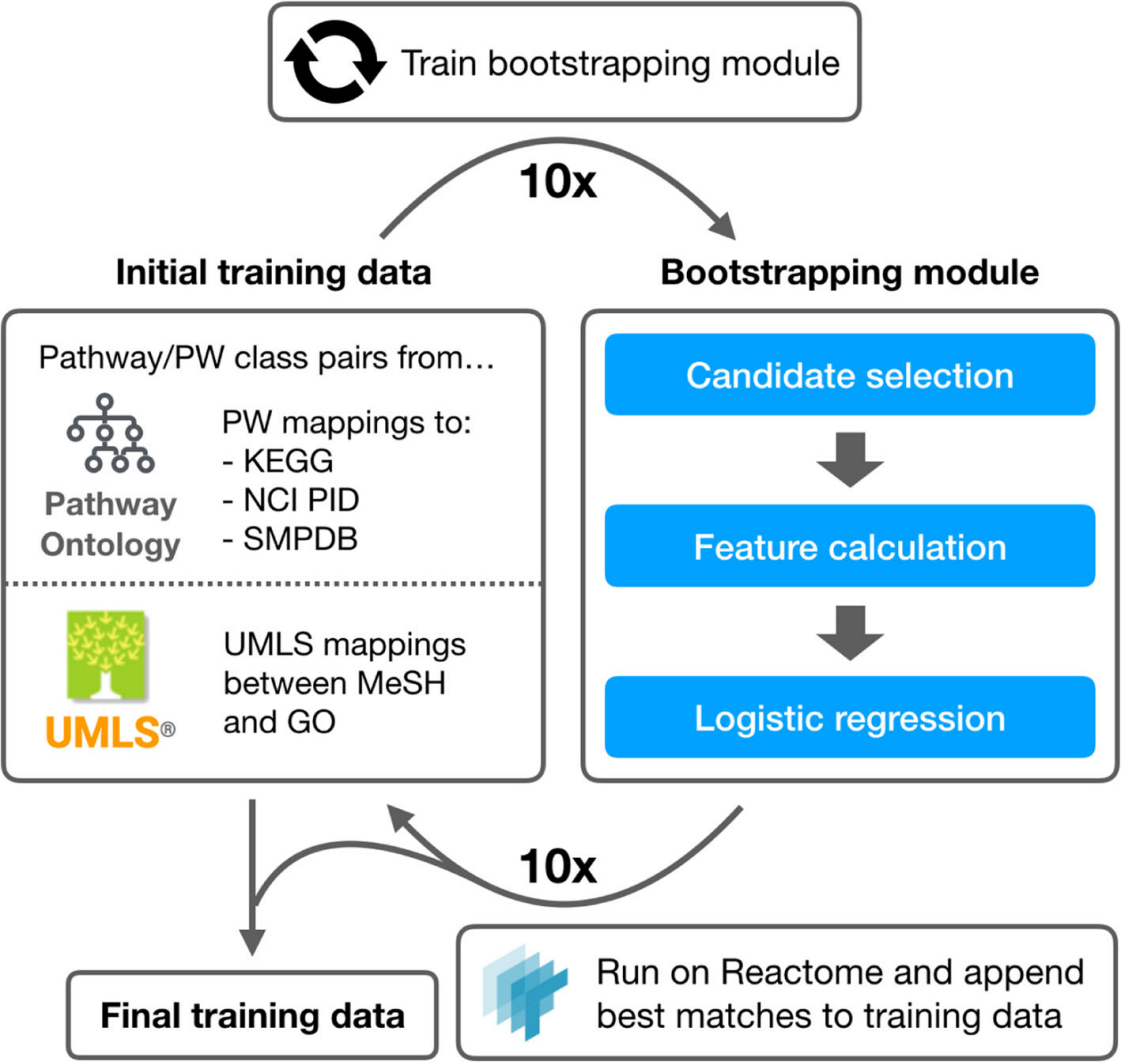
Bootstrapping procedure. The initial training data is derived from existing PW mappings and UMLS mappings between MeSH and GO. A simple logistic regression model is trained on this data and used to bootstrap training samples from Reactome. The best matches between Reactome pathways and PW classes are added to the training data set over 10 iterations to generate a final training data set

To further boost training data, we extract high probability positive matches between the PW and pathways from Reactome. Including training examples from Reactome adapts the predictive model to the specifics of the Reactome database and we can expect an improvement in prediction quality. A bootstrapping procedure (Fig. 2) is used to iteratively train a predictive model and append its highest likelihood predictions to the training data [27]. We employ a simple logistic regression model using manually engineered lexical similarity features. The features we use are:

- Normalized absolute value percent word token number difference
- Word token Jaccard index
- Character *n*-gram Jaccard index for *n*=3, 4, 5

For each bootstrapping iteration, we train a logistic regression model over the training data. We run this trained model over the PW and Reactome, generating a set of predicted PW classes for each pathway in Reactome. The top and bottom 0.25% of predictions are added to the training data as respective positive and negative training examples for the following iteration. We iteratively train the bootstrapping module 10 times, generating 730 positive and 720 negative training samples from Reactome. A cursory review of the added training samples revealed good quality matches (88% correct at iteration 10), where most of the matches could be considered “low-hanging fruit,” with pathway and PW class names that match well based on string similarity alone. Incorrect matches have very close semantic relationships, such as the Reactome pathway for RNA polymerase II transcription matching to the PW class for RNA polymerase I transcription.

### Neural network

We constructed two neural network models for processing pathway names and pathway definitions. We begin by describing the pathway name model.

Each pathway name is represented using pre-trained word embeddings. For each word token, we concatenate a 100-dimensional *word2vec* [28] vector and a 100-dimensional *fasttext* [29] vector, generating a 200-dimensional word vector. Both *word2vec* and *fasttext* embeddings are trained on Pubmed Central full-length journal articles. *Word2vec* tends to capture the semantic context of a word and *fasttext* its internal structure (prefixes, suffixes etc), so combining the two allows us to capture information about both the meaning and appearance of a word.

The pathway name is treated as a bag of word embeddings; the word-level embeddings of each word token in the name are summed, generating a pathway name embedding: a 200-dimensional vector. A PW class name embedding is generated from the PW class name in a similar fashion. These two embeddings are concatenated and input into a decision network consisting of two fully connected neuron layers. A sigmoid function processes the output of this network, producing a final similarity score between 0 and 1, which is thresholded to determine the binary class output.

**Fig. 3:**
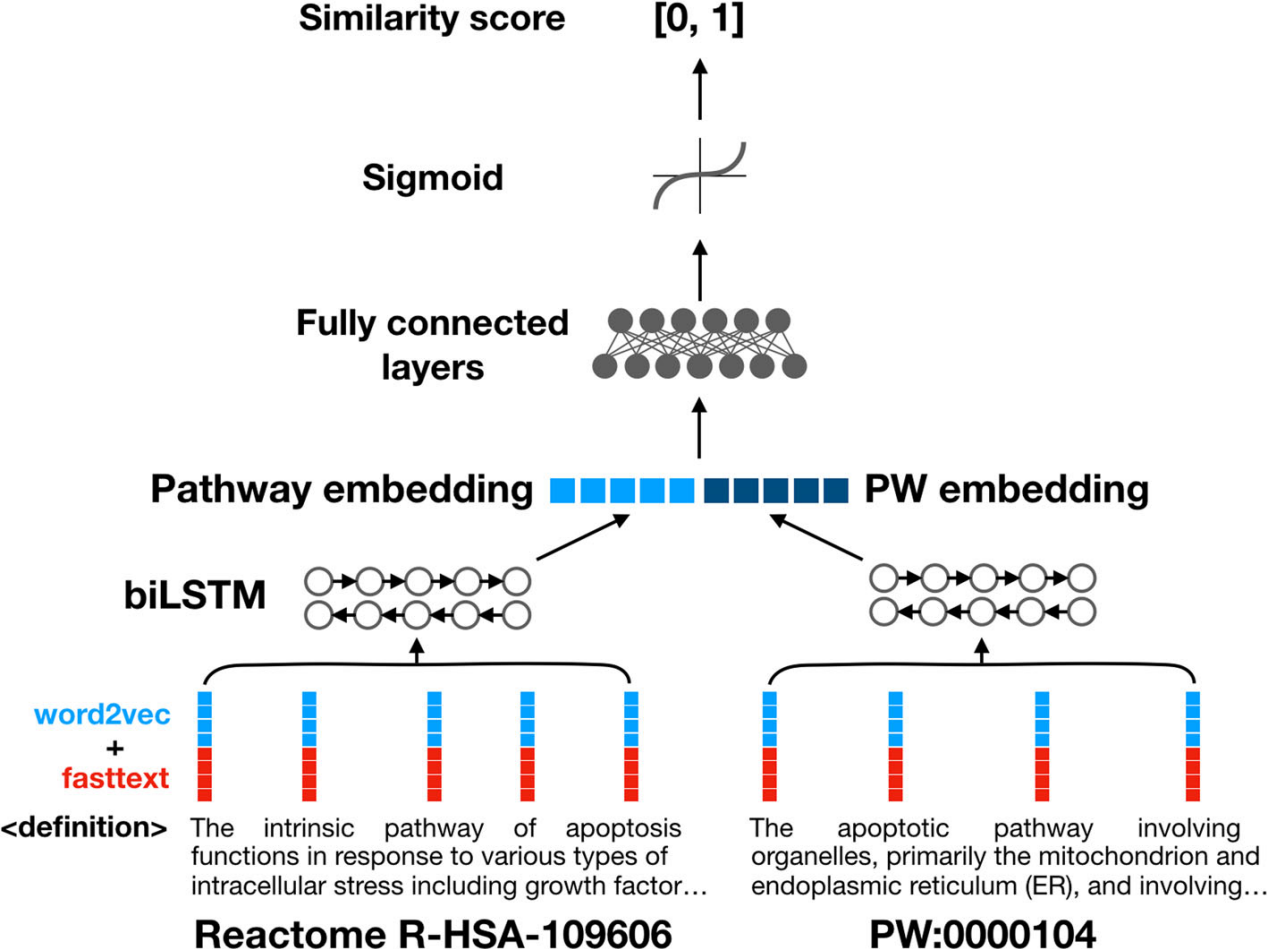
Architecture of neural network model. The neural network computes similarity between a pathway definition and a PW class definition. A bidirectional LSTM is used to encode the definition texts. This example shows the definition for Reactome pathway R-HSA-109606 and PW class PW:0000104 being encoded and compared in the neural network

Pathway definitions consist of longer pieces of text with many internal relationships (see Fig. 3 for examples). Instead of bag-of-embeddings, a bidirectional long-short term memory (LSTM) network is used to capture more semantic information [30]. The hidden layers at both ends of the LSTM are concatenated to produce a pathway definition embedding vector. The pathway definition embedding and PW class definition embedding vectors are then concatenated and input into a decision network of fully connected neuron layers. Similarly, an output score between 0 and 1 is generated as output using a sigmoid function. Figure 3 shows the network architecture of the definition model; the name model uses bag-of-embeddings networks in lieu of the LSTMs.

The final training data are split into a training (90%) and development (10%) set. The models are trained to minimize the binary cross-entropy loss with respect to the training labels. We use the development set to optimize model training for recall, because we are more concerned about deriving all possible matches rather than all certain matches.

### Combining predictions

The trained neural networks are used to predict mappings between Reactome and the PW. For each pathway in Reactome, the candidate selector selects the top 20 PW classes, generating up to 20 candidate pairs. For each candidate pair (*N*,*M*), where *N* is a pathway from Reactome and *M* a class from PW, *N* has names *N*_*name*_={*n*_1_,*n*_2_,...,*n*_*p*_} and *M* has names *M*_*name*_={*m*_1_,*m*_2_,...,*m*_*q*_}. These names are formed into unique name pairs by taking the Cartesian product of *N*_*name*_ and *M*_*name*_. Each pair of names (*i*,*j*) is fed into the name neural network model, producing a set of name similarity scores:

2 $$ \mathbf{S_{name}} = \{s_{ij} \quad \vert \quad (i, j) \, \epsilon \, \mathbf{N_{name}} \times \mathbf{M_{name}}\}  $$

Each score *s*_*ij*_ is the similarity between the pathway name *i* and PW class name *j*.

If the Reactome pathway has a definition, then the definition texts of the pathway and PW class are fed into the definition neural network model, yielding a single similarity score *S*_*def*_. A final similarity score is produced by combining and weighting the name and definition similarities:

3 $$ S_{total} = 0.75 \max{(\mathbf{S_{name}})} + 0.25 S_{def}  $$

The weights of max(*S*_*name*_) and *S*_*def*_ are selected to favor name similarity because in many cases, there is a lack thereof or non-specific definition in Reactome. More optimal weights are likely to exist, but we do not explore them in this work due to limited resources for evaluation. Matching PW classes with *S*_*total*_ above a threshold of 0.25 are output by the predictive model.

### Evaluation of model results

For evaluation, a 5% subset of pathways from Reactome were randomly selected, a total of 111 pathways out of 2222. For this subset, all output predictions from both the BOW and NN model were extracted and presented to two curators for manual review. Output predictions were presented to curators after first grouping by Reactome pathway and then sorting the PW classes within each group by similarity score. A separate subset of 211 class recommendations produced by the NN model was also evaluated by both curators, allowing us to determine inter-rater agreement.

Curators were asked to perform the following task on each selected subset: for each Reactome pathway-PW class pair, grade the pair as y(es)/n(o)/r(elated), where y(es) indicates an exact match, n(o) indicates an incorrect match, and r(elated) indicates that although the pair is not an exact match, the pathway is related to the PW class (maps to parent, child, or sibling classes). Two metrics are computed over the labeled results, precision per mapping (*ppm*) and recall per pathway (*rpp*). The *ppm* is defined as the ratio of pathway-PW class pairs rated y(es) or r(elated) over all pairs rated. It is a measure of how correct the models are for each recommendation produced. The *rpp* is defined as the number of pathways for which at least one y(es) or r(elated) PW class is recommended over the total number of pathways. It is a measure of how successful the algorithm is at making at least one successful recommendation for each pathway. We also report the yield of both models over all Reactome pathways. The yield indicates the percentage of pathways receiving *any* recommended PW mappings.

For each Reactome pathway, curators also selected the correct mapping, either from among the predicted PW class matches, or from elsewhere in the PW. These mappings are added to the PW for future release. In cases where a correct mapping is not predicted by our model, curators must determine whether a new class or relation needs to be added to accommodate the Reactome pathway in question.

## Results

The model was used to generate PW mapping recommendations for Reactome human pathways. The BOW model yielded 4122 mapping suggestions for 2222 Reactome pathways. The NN model produced 10,952 suggestions for the same pathways. Table 2 shows example NN predictions generated for the Reactome human apoptosis pathway, R-HSA-109581, of which there is no direct name-matched class in the PW. The predictions show that the predictive model is able to retrieve PW classes that are similar to the Reactome pathway in both name and content. The top predicted matches are those describing the apoptotic process, followed by those describing related processes in immune response and cell death. Of these recommended PW classes, the correct match is to PW:0000009, the apoptotic cell death pathway, the second ranked PW class recommended by the predictive model. This PW class was selected by curators as the correct PW mapping for R-HSA-109581.

**Table 2:** Top ranked predicted mappings for Reactome pathway R-HSA-109581, “Apoptosis”

	PW ID	PW class name	Beginning of definition text
1	PW_0000104	intrinsic apoptotic pathway	The apoptotic pathway involving organelles, primarily the mitochon...
2	PW_0000009	apoptotic cell death pathway	Apoptosis is a programmed cell death pathway that is characterized by...
3	PW_0000106	extrinsic apoptotic pathway	The apoptotic pathway involving the death receptors mediated route of...
4	PW_0000718	p53 signaling pathway	p53 transcription factor is a tumor suppressor frequently mutated in...
5	PW_0000124	cellular detoxification pathway	A pathway triggered by exogenous or endogenous elements, compounds...
6	PW_0000823	humoral immunity pathway	Humoral immunity is mediated by antibodies secreted by the B cell...
7	PW_0000824	cell-mediated immunity pathway	Cell-mediated immune response pathways are carried out by T cell...
8	PW_0000499	nuclear factor kappa B signaling pathway	NF-kB signaling plays an essential role in the mammalian immune...
9	PW_0000680	altered extrinsic apoptotic pathway	*<no definition >*
10	PW_0000233	tumor necrosis factor mediated signaling pathway	Tumor necrosis factor (Tnf) signaling plays pivotal roles in immunity...

Two RGD curators (GTH and MT) conducted a reproducibility review of the predictions. Table 3 shows the results of the reproducibility analysis. Review of 211 class recommendations showed a 0.73 agreement between two reviewers for each mapping (Cohen’s kappa for three classes (y/n/r) = 0.56).

**Table 3:** Inter-rater agreement for mapping labeling task

	Rater #1			
Rater #2	y(es)	r(elated)	n(o)	Totals
y(es)	24	8	0	32
r(elated)	0	69	4	73
n(o)	0	46	60	106
Totals	24	123	64	211

A comparison of BOW and NN models is provided in Table 4. Curators reviewed 243 mapping recommendations produced by the BOW model for 111 randomly sampled pathways, and 660 recommendations produced by the NN model for the same 111 pathways. The BOW model had significantly lower yield compared to the NN model (BOW: yield = 0.50; NN: yield = 0.80). Although the BOW model had higher precision than the NN model (BOW: *ppm* = 0.49; NN: *ppm* = 0.39), it also had correspondingly lower recall (BOW: *rpp* = 0.42; NN: *rpp* = 0.78). Overall, the NN model provided more opportunities for selecting an appropriate mapping. Perhaps combining the outputs of both models could produce better coverage with higher precision.

**Table 4:** Comparison of BOW and NN model predictions

Model	Precision (*ppm*)	Recall (*rpp*)	Yield
BOW	0.49	0.42	0.50
NN	0.39	0.78	0.80

Precision and recall are calculated from a 5% sample of Reactome pathways; yield is calculated over all Reactome pathways

A number of pathways did not receive relevant suggestions via either model. Reactome, in particular, contains very specialized regulatory pathway representations that do not currently have corresponding classes in the PW. Some portions of the PW class hierarchy, such as those describing the immune system and cellular signaling, may require further development. For example, several Reactome pathways dealing with interferon-mediated immunity, such as R-HSA-1834941 (“STING mediated induction of host immune responses”) or R-HSA-918233 (“TRAF3-dependent IRF activation pathway”) do not have corresponding pathway classes in the PW. The PW contains classes for type I (PW:0000895) and type II (PW:0000896) interferon signaling pathways, and has several subclasses describing signaling pathways related to innate immune response (PW:0000819), but none of these existing classes are suitable for describing the functions represented by the example Reactome pathways. The PW may need to add either more granular pathway classes, or introduce properties such as *regulates* or *related_to* to annotate the relationships described above and found throughout pathways from Reactome.

The above methods can also be applied to other pathway databases. As a test of generalizability, we ran the trained predictive model over pathways from HumanCyc and WikiPathways, generating predicted mappings to the PW. The NN model produced 1199 recommendations for 217 HumanCyc pathways and 1652 recommendations for 351 WikiPathways pathways. These recommendations have yet to be reviewed by curators, but can provide a helpful starting point when mapping pathways from these other databases to the PW. Early inspection of the results suggest that similar pathways between these databases receive mappings to similar or the same PW classes. For example, Table 5 shows the top pathways from Reactome, HumanCyc, and WikiPathways that the NN model associated with PW:0000029, the “fatty acid biosynthetic pathway.” Although imperfect, the recommendations are largely relevant. Note that fewer pathways from HumanCyc and WikiPathways are associated with this PW class; this is due to both the smaller size of the HumanCyc and WikiPathways databases, but also the granularity of represented pathways.

**Table 5:** Top pathways predicted to map to PW:0000029 ("fatty acid biosynthetic pathway")

HumanCyc	Reactome	WikiPathways
PWY-5966: fatty acid biosynthesis initiation II	R-HSA-77288: mitochondrial fatty acid beta-oxidation of unsaturated fatty acids	WP357: Fatty Acid Biosynthesis
PWY-5143: fatty acid activation	R-HSA-77289: Mitochondrial Fatty Acid Beta-Oxidation	
	R-HSA-390247: Beta-oxidation of very long chain fatty acids	
	R-HSA-75105: Fatty acyl-CoA biosynthesis	
	R-HSA-500753: Pyrimidine biosynthesis	
	R-HSA-8978868: Fatty acid metabolism	

Curator-selected mappings between Reactome and PW classes can be used as an additional source of training data for improving the predictive model. As the quantity of high-quality training data increases, our predictive model should improve, helping to further reduce the curatorial burden of mapping other pathway databases to the PW.

## Discussion

We have described our efforts to incorporate a predictive classifier into the PW curation pipeline for generating mappings between pathway databases and the PW. Results demonstrate that our model is able to recommend relevant PW class mappings for pathways. By automatically inferring high-likelihood mappings between pathways and PW classes, we hope to reduce the burden on curators.

Our decisions maximalize annotation success based on the curation pipeline described in Figure 1. For example, we bias the NN model during training to maximize recall. This is desirable because we have the luxury of manual curatorial review as a gatekeeper to annotation. When operating in situations without manual review, it may be more desireable to bias the model towards maximizing metrics such as precision or accuracy.

The mappings we generated between Reactome pathways and PW classes contribute to our overall goal of pathway data organization and integration. By organizing pathways from different databases under a single unifying ontology, we can understand how pathway data from different databases relate to one another. We can use the PW class hierarchy to reduce redundancy among pathway datasets by merging pathways under each PW class into normalized pathways. Normalized pathways may have better interpretability due to the class boundaries and relationships provided by the ontology.

As described in previous publications, we face many challenges to pathway data integration, such as 1) the usage of different pathway organizational schemes by different databases, 2) incomplete or inconsistent description of pathway-subpathway relationships, as well as 3) differences in identifier and semantic choices in representing pathway data among the various source databases [6, 7, 12, 31]. Using a unifying ontology for organization at the pathway level will ameliorate the first two of these challenges. To address the third, we have demonstrated methods of entity disambiguation and graph alignment capable of aligning pathways even in the presence of identifier or semantic differences [32]. In this prior work, we explored lexical and topological techniques for pathway alignment. These pathway alignment techniques should be able to handle many of the described representational differences when merging pathways.

### Limitations

The current mapping prediction algorithm uses pathway name and definition information (and to some extent, the names of parent and child pathways and PW classes, through the candidate selector) to match pathways with PW classes. The algorithm does not incorporate the pathway content itself: the graph of entities and relationships that describe biological function. By incorporating textual descriptions of pathways, we believe we capture most of the important entities and relationships in a pathway. Explicit information on pathway member entities were left out of the current mapping algorithm due to concern about increasing the size of the predictive model, and challenges in representing this information as model input. How to include this additional information in prediction is an open research question.

Pathway databases are all different, each with its own strengths and limitations. What works for Reactome may not apply directly to all other pathway databases. Although we have demonstrated the ability to apply the predictive algorithm to HumanCyc and WikiPathways, we have not yet evaluated the resulting predictions. We have also not evaluated how newly generated Reactome mappings may benefit the detection of mappings between other pathway databases and the PW. Because these other databases emphasize different aspects of pathway data (e.g., the BioCyc databases contain more information on conserved metabolic pathways between species), they may require alternate curatorial choices for selecting appropriate mappings and for handling pathways without matching PW classes. These decisions will need to be explored in a further study of generalizability.

We would also like to explore how our predictive algorithm may apply to other ontologies and datasets. The authors believe that the design of the bootstrapping algorithm and the neural network may need significant adaptation to work in other biomedical domains. The current predictive algorithm depends on the presence of existing mappings that can be extracted and used as training data. In cases where there is no access to pre-existing mappings between data and ontology, a simple machine learning model similar to that used in the bootstrapping procedure may be more fitting.

### Future work

RGD annotators are reviewing the remaining mapping recommendations for Reactome pathways and adding new mappings into the PW. Reviewers are also annotating pathways based on predictions for BioCyc and WikiPathways pathways. The predictive model will be retrained incorporating the additional mappings generated by this project. Upon completion of the overall mapping project, the PW will contain mappings to six pathway databases: the three that precede the developments described in this paper (KEGG, NCI-PID, and SMPDB), and three new pathway databases (BioCyc, Reactome, and WikiPathways).

As alluded to earlier, some pathways from these databases do not have direct correspondences in the Pathway Ontology. In some cases, pathways representing processes at fine granularity can only be mapped to more general PW classes. These observations suggest that semi-automated ontology annotation prediction could play a helpful role in ontology completion or ontology development. We are investigating the differences between poor recommendation quality (failure of the model) and the lack of appropriate recommendations (insufficient representation in the ontology). In future work, we would like to produce a model that distinguishes between these two situations.

### Conclusion

Pathway representations are critical for modeling and understanding the physiological processes underlying both normal and disease health states, but a lack of understanding of the relationships between pathways of different provenance undermine their collective usability. Combining the data from different pathway databases using a unifying ontology could address many of these issues. We demonstrate in this article the design, implementation and evaluation of a computationally-assisted pipeline for mapping Reactome pathways to classes in the Pathway Ontology. Initial results of the classification model show promise, highlighting a number of pathway instance to PW class mappings that should be assessed by curators. We are working towards improving the quality and quantity of these mapping recommendations, as manual curation continues over the results for Reactome and other pathway databases. Following the completion of pathway mapping, we will proceed by aligning pathways grouped together under each PW class, generating normalized pathway representations. Merging pathway instances along ontological class lines will produce non-redundant yet interpretable pathways for use in secondary statistical analysis.

## Data Availability

An implementation of the model is available at https://www.github.com/lucylw/pathhier/. The Pathway Ontology is available on BioPortal at https://bioportal.bioontology.org/ontologies/PW.
